# Interactions between C8orf37 and FAM161A, Two Ciliary Proteins Essential for Photoreceptor Survival

**DOI:** 10.3390/ijms231912033

**Published:** 2022-10-10

**Authors:** Yu Liu, Jinjun Chen, Rachel Sager, Erika Sasaki, Huaiyu Hu

**Affiliations:** 1 Center for Vision Research, Departments of Neuroscience and Physiology and of Ophthalmology and Visual Sciences, Upstate Medical University, Syracuse, NY 13210, USA; 2 College of Bioscience and Biotechnology, Hunan Agricultural University, Changsha 410128, China; 3 Department of Marmoset Biology and Medicine, Central Institute for Experimental Animals, Tonomachi, Kawasaki 210-0821, Kanagawa, Japan

**Keywords:** retinal degeneration, Bardet-Biedl syndrome, C8orf37, FAM161A, cone–rod dystrophy, retinitis pigmentosa

## Abstract

Mutations in C8orf37 cause Bardet-Biedl syndrome (BBS), retinitis pigmentosa (RP), and cone–rod dystrophy (CRD), all manifest in photoreceptor degeneration. Little is known about which proteins C8orf37 interacts with to contribute to photoreceptor survival. To determine the proteins that potentially interact with C8orf37, we carried out a yeast two-hybrid (Y2H) screen using C8orf37 as a bait. FAM161A, a microtubule-binding protein localized at the photoreceptor cilium required for photoreceptor survival, was identified as one of the preys. Double immunofluorescence staining and proximity ligation assay (PLA) of marmoset retinal sections showed that C8orf37 was enriched and was co-localized with FAM161A at the ciliary base of photoreceptors. Epitope-tagged C8orf37 and FAM161A, expressed in HEK293 cells, were also found to be co-localized by double immunofluorescence staining and PLA. Furthermore, interaction domain mapping assays identified that the N-terminal region of C8orf37 and amino acid residues 341-517 within the PFAM UPF0564 domain of FAM161A were critical for C8orf37–FAM161A interaction. These data suggest that the two photoreceptor survival proteins, C8orf37 and FAM161A, interact with each other which may contribute to photoreceptor health.

## 1. Introduction

Mutations in nearly 300 genes have been identified as causes of retinal degeneration. Abnormal C8orf37 have been found in patients with Bardet-Biedl syndrome (BBS) [1,2]. BBS (MIM209900) is characterized by photoreceptor degeneration, obesity, digit anomalies, genito-urinary anomalies, and cognitive deficits. This autosomal recessive disorder is caused by mutations in over 20 genes that are implicated in the function of primary cilia. Mutations in C8orf37 can also cause non-syndromic retinal degeneration including cone–rod dystrophy (CRD, MIM 120,970, cone–rod dystrophy 16 (CORD16) [3,4,5]) and retinitis pigmentosa (RP, MIM 268,000, retinitis pigmentosa 64 (RP64) [3,6,7,8,9]). Retinitis pigmentosa consists of a group of blinding diseases that are genetically heterogeneous. Amongst all inherited retinal degenerative diseases, RP is the most common form and is characterized by an initial loss of rod photoreceptors followed by loss of cone photoreceptors. In cone–rod dystrophy, cone photoreceptors are predominantly affected. Loss of vision in these diseases is usually progressive and severe, with no effective treatment.

Human C8orf37 is a ubiquitously expressed protein with 207 amino acid residues and has no identified structural domains. How C8orf37 protein contributes to photoreceptor survival is unknown. Previous research suggests that C8orf37 may be involved in the function of primary cilia. C8orf37 immunoreactivity is found at the ciliary base in hTert-RPE1 cells as well as the ciliary base and inner segments of mouse photoreceptors [6]. Knock-down in zebrafish reduces the formation of cilia in the Kupffer’s vesicle, affecting the development of left–right asymmetry [1]. We hypothesize that C8orf37 may interact with other ciliary proteins. In an effort to identify molecules that interact with C8orf37 and to shed light on how C8orf37 contributes to photoreceptor health, we carried out a yeast two-hybrid (Y2H) screen using C8orf37 as a bait. FAM161A was identified as a potential interaction candidate. Previous studies have implicated FAM161A in autosomal-recessive retinitis pigmentosa 28 (RP28) [10,11]. FAM161A is a microtubule-binding protein expressed throughout photoreceptor inner segments and enriched at the base of photoreceptor connecting cilia and basal bodies [12,13]. It interacts with other ciliary proteins such as lebercilin, CEP290, OFD1, and SDCCAG8 through its C-terminal region [12]. Our results indicated that amino acid residues 1–75 of C8orf37 also interacted with the C-terminal region of FAM161A, specifically, the Pfam UPF0564 domain.

## 2. Results

### 2.1. Co-localization of C8orf37 and FAM161A in the Marmoset Retina

A yeast two-hybrid screen of human retinal library, using full length human C8orf37 as a bait, identified 28 potential interactions (Table 1). Two of these genes have been implicated in retinal degeneration. Mutations in transcription factor CRX are known to cause autosomal dominant cone–rod dystrophy type 2, Leber congenital amaurosis type 7 and autosomal dominant retinitis pigmentosa “https://web.sph.uth.edu/RetNet/disease.htm (accessed on 1 October 2022)”. Interestingly, the prey FAM161A is a microtubule-binding protein enriched at the ciliary base of photoreceptors [12,13] and has been implicated in retinitis pigmentosa 28 [10,11]. The Y2H screening captured a fragment of FAM161A that corresponded to aa 317–553, which lies within its PFAM UPF0564 domain. To evaluate whether C8orf37 and FAM161A proteins in photoreceptors were co-localized, we first carried out double immunofluorescence staining of common marmoset retinal sections using rabbit anti-C8orf37 or rabbit anti-FAM161A with mouse anti-acetylated α-tubulin, an axoneme marker. We chose the marmoset retina for evaluating their co-localization because the commercially available antibodies were raised against human proteins, and thus more likely to recognize primate antigens than those of the mouse.

**Figure 1:**
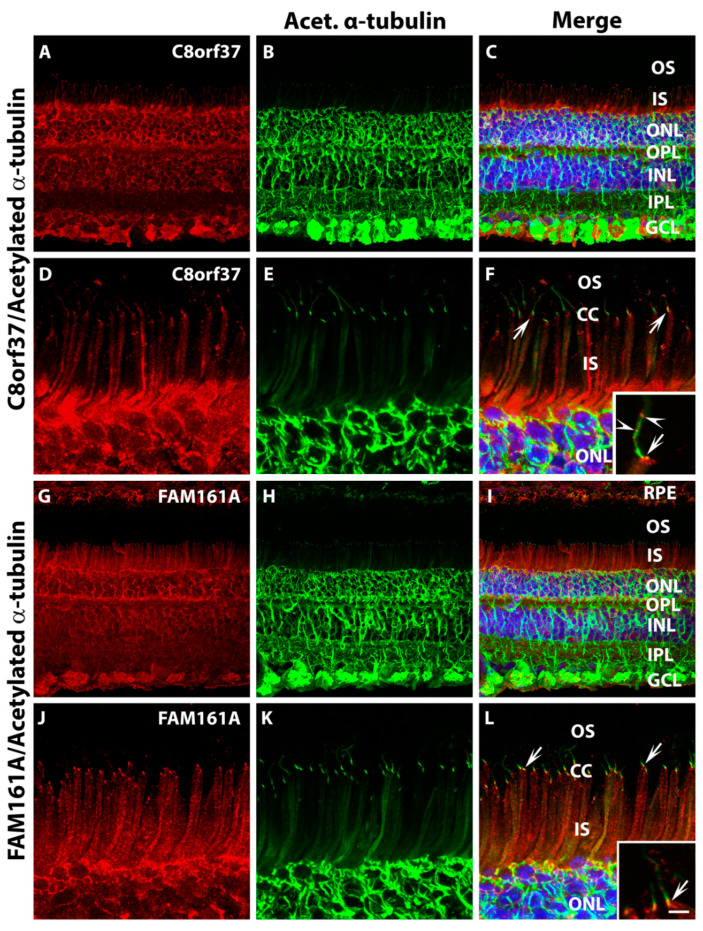
C8orf37 and FAM161A were widely expressed in the marmoset retina. Marmoset retinal sections were double-immunostained with anti-C8orf37 (red) and anti-acetylated α-tubulin (green) (**A**–**F**) or anti-FAM161A and acetylated α-tubulin (green) (**G**–**L**). The sections were counterstained with DAPI (blue). Images (**A**–**F**) show C8orf37 and acetylated α-tubulin double staining. C8orf37 immunoreactivity was observed in the inner segment layer, outer nuclear layer, inner nuclear layer, and ganglion cell layer. C8orf37 immunoreactivity puncta was also observed at the base of acetylated α-tubulin-labeled cilia (arrows in **F**), as well as along the cilia (arrowheads). Images (**G**–**L**) show FAM161A and acetylated α-tubulin double staining. FAM161A immunoreactivity was widely distributed in the retina. Its puncta were also enriched at the base of cilia (arrows in **L**) and along the cilia. Abbreviations: GCL, ganglion cell layer; INL, inner nuclear layer; IPL, inner plexiform layer; IS, inner segment; ONL, outer nuclear layer; OPL, outer plexiform layer; OS, outer segment. Scale bar: 20 μm for (**A**–**C**) and (**G**–**I**); 6 μm for (**D**–**F**) and (**J**–**L**); 3 μm for insets in (**F**) and (**L**).

C8orf37 protein has been previously reported to be highly enriched at the ciliary base at the junction between the outer and inner segments of photoreceptors [6]. In this study, we used an anti-C8orf37 antibody with immunofluorescence pattern identical to turboGFP fluorescence in HEK293 cells transfected with a C8orf37-turboGFP fusion construct (Supplementary Figure S1A–C). This antibody also recognized the same band as anti-turboGFP on Western blots of C8orf37–turboGFP-transfected cell lysate, supporting antibody specificity (Supplementary Figure S1D). C8orf37 immunoreactivity was widely distributed in the neural retina with strong immunofluorescence reactivity in the outer nuclear layer (ONL), inner nuclear layer (INL), and ganglion cell layer (GCL) (Figure 1A,C). Anti-acetylated α-tubulin staining was used to identify photoreceptor axonemes (Figure 1B,E,H,K). C8orf37 immunoreactivity was enriched at the inner segment, including the ciliary base (Figure 1D,F, arrows) and along the length of the photoreceptor axoneme (Figure 1F, arrowheads). Similarly, FAM161A immunoreactivity was also widely distributed in the neural retina (Figure 1G,I,J,L). As reported in mouse photoreceptors [12,13], FAM161A immunoreactivity was also enriched in the inner segment and highly enriched at the ciliary base (Figure 1L, arrows).

**Figure 2:**
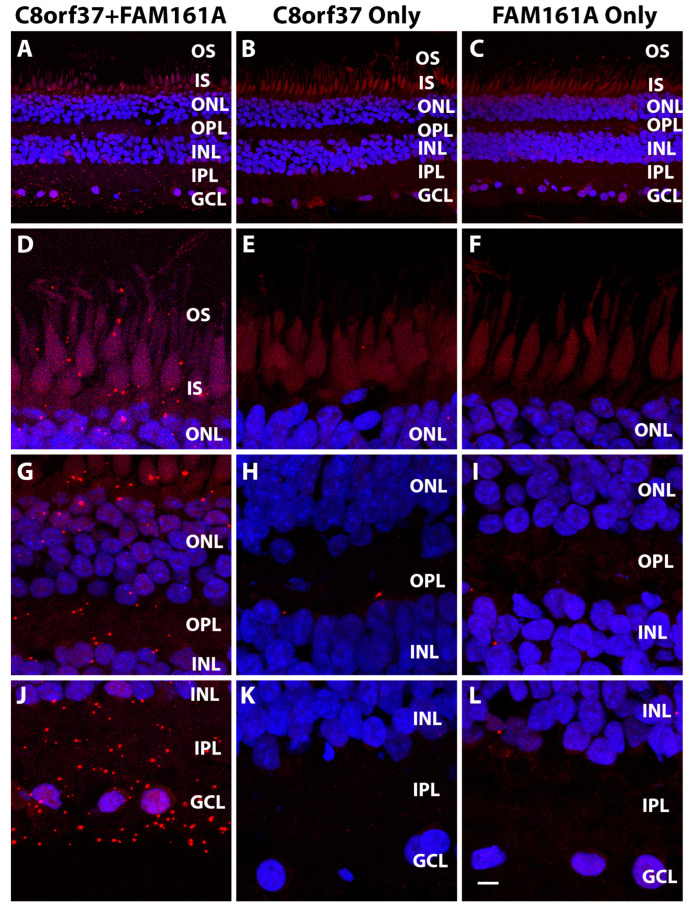
Proximity ligation assay of C8orf37 and FAM161A in the marmoset retina. Proximity ligation assay with Duolink In Situ Probemaker PLUS-labeled anti-C8orf37 and Duolink In Situ Probemaker MINUS-labeled FAM161A was performed on marmoset retinal sections, showing: (**A**,**D**,**G**,**J**) PLA with both anti-C8orf37-PLUS and anti-FAM161A-MINUS probes; (**B**,**E**,**H**,**K**) Anti-C8orf37-PLUS probe alone; (**C**,**F**,**I**,**L**) Anti-FAM161A-MINUS probe alone. Note the PLA signals observed in the retina when both probes were used. Few signals were observed when anti-C8orf37 and anti-FAM161A were used alone. Scale bar in L: 20 μm for (**A**–**C**); 6 μm for (**D**–**L**).

**Figure 3:**
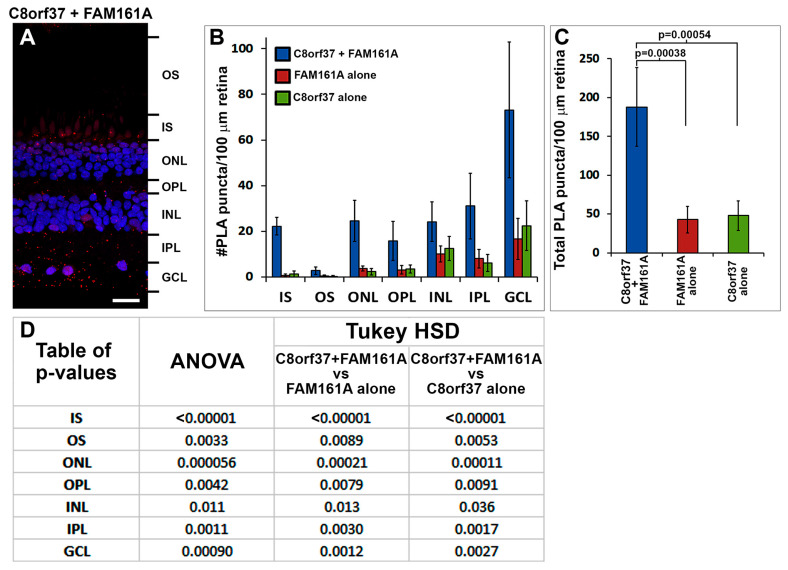
Proximity ligation assay quantification for C8orf37 and FAM161A in the marmoset retina. Confocal images of the retina were divided into layers (example is shown in (**A**)). PLA puncta in each layer were counted from images of 5 stained sections from one marmoset eye, showing: (**B**,**C**) the numbers of PLA puncta in each layer of the retina, as well as the total number of PLA puncta across all layers of the retina. Error bars represent standard error of the mean (SEM). (**D**) Table of *p*-values from ANOVA and Tukey HSD post-hoc tests. The number of PLA puncta was significantly increased when PLA was performed with both probes compared with when anti-C8orf37-PLUS or anti-FAM161A-MINUS probes were used alone. Scale bar in (**A**): 20 μm.

To evaluate whether C8orf37 and FAM161A were co-localized, we performed proximity ligation assays by conjugating anti-C8orf37 and anti-FAM161A antibodies with Duolink™ In Situ Probemaker PLUS probes and Duolink™ In Situ Probemaker MINUS probes (Sigma Aldrich, Burlington, MA, USA), respectively. When both C8orf37-conjugated PLUS probes and FAM161A-conjugated MINUS probes were used, PLA puncta were found throughout the neural retina (Figure 2A,D,G,J). When PLA was performed with either C8orf37–PLUS or FAM161A-MINUS probes alone, PLA puncta were significantly reduced (Figure 2B,C,E,F,H,I,K,L). We quantified the PLA puncta, comparing their numbers between C8orf37/FAM161A double probe assays and C8orf37-only and FAM161A-only single probe assays (Figure 3). The numbers of PLA puncta in every retinal layer were higher when both probes were used than when single probes were used (ANOVA followed by post-hoc Tukey HSD, Figure 3D). There was no statistically significant difference between the C8orf37-only and FAM161A-only single probe control assays (Figure 3B,C). Together, these data indicate that C8orf37 and FAM161A immunoreactivity was co-localized in the marmoset retina, including photoreceptors.

### 2.2. Co-Localization of Epitope-Tagged C8orf37 and FAM161A in Transfected HEK293 Cells

**Figure 4:**
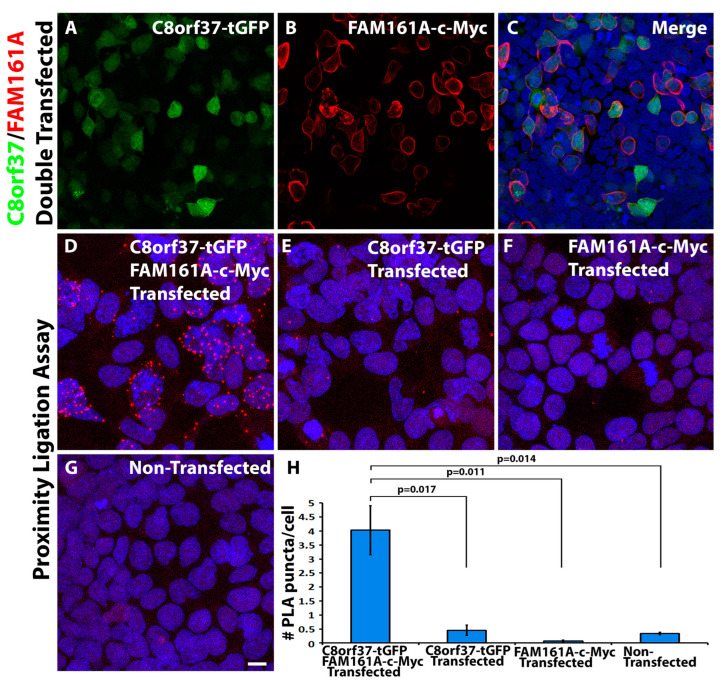
Overexpressed C8orf37 and FAM161A were co-localized. HEK293 cells were transfected with pCMV-C8orf37-tGFP and pCMV-FAM161A-c-Myc expression constructs. The cells were immunostained with anti-c-Myc (red) (**A**–**C**) or processed for proximity ligation assay using anti-tGFP and anti-c-Myc antibodies (**D**–**G**). Images (**A**–**C**) show pCMV-C8orf37-tGFP and pCMV-FAM161A-c-Myc double transfected cells. HEK293 cells were efficiently transfected with both plasmids. Image (**D**) shows that PLA puncta were frequently observed in cells double transfected with pCMV-C8orf37-tGFP and pCMV-FAM161A-c-Myc. Images (**E**–**G**) show that PLA puncta were significantly reduced when the cells were single-transfected with pCMV-C8orf37-tGFP (**E**), or pCMV-FAM161A-c-Myc (**F**), or non-transfected (**G**). Image (**H**) shows quantification of PLA puncta three-repeat transfections. ANOVA followed by post-hoc Student’s t-test with Bonferroni correction. Error bars represent SEM. Scale bar in (**G**): 20 μm.

Since antibodies may recognize nonspecific antigens in immunostaining, some of the PLA puncta detected in the marmoset retina might have arisen from non-specific binding of anti-C8orf37 and anti-FAM161A. Thus, we sought to confirm the interaction with epitope-tagged C8orf37 and FAM161A heterologously expressed in HEK293 cells using epitope-specific antibodies. We obtained turboGFP-tagged human C8orf37 and c-Myc-tagged human FAM161A expression constructs from Origene Technologies (Rockville, MD, USA), pCMV-C8orf37-tGFP and pCMV-FAM161A-c-Myc. HEK293 cells were co-transfected with these epitope-tagged constructs. GFP fluorescence and immunofluorescence staining with c-Myc antibody indicated efficient transfection for both constructs (Figure 4). In double transfected cells, overlapping immunofluorescence was observed (Figure 4A–C). We then carried out proximity ligation assays with antibodies against turboGFP and c-Myc. PLA puncta were frequently observed in double-transfected cells (Figure 4D). By contrast, the numbers of PLA-positive puncta observed in cells transfected with pCMV-C8orf37-tGFP alone, in cells transfected with pCMV-FAM161A-c-Myc alone, or in non-transfected cells were significantly reduced (Figure 4E–H). These results suggested that overexpressed C8orf37 and FAM161A can be co-localized.

### 2.3. N-Terminal Region of C8orf37 Interacted with FAM161A

**Figure 5:**
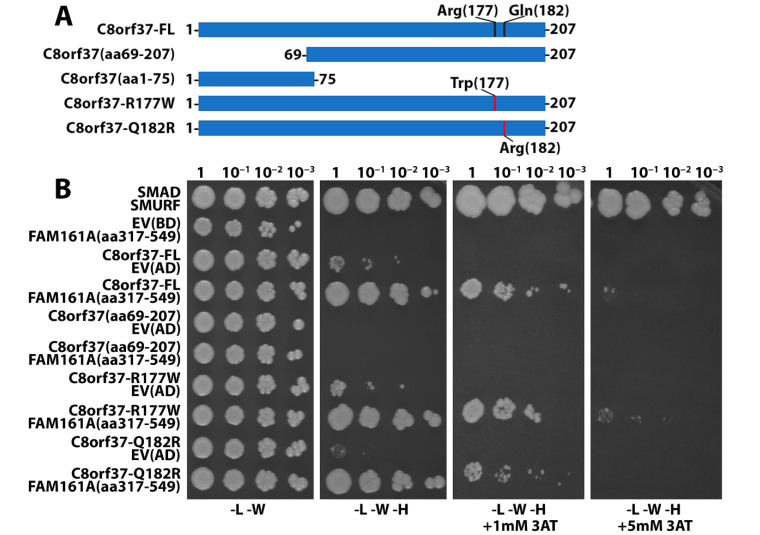

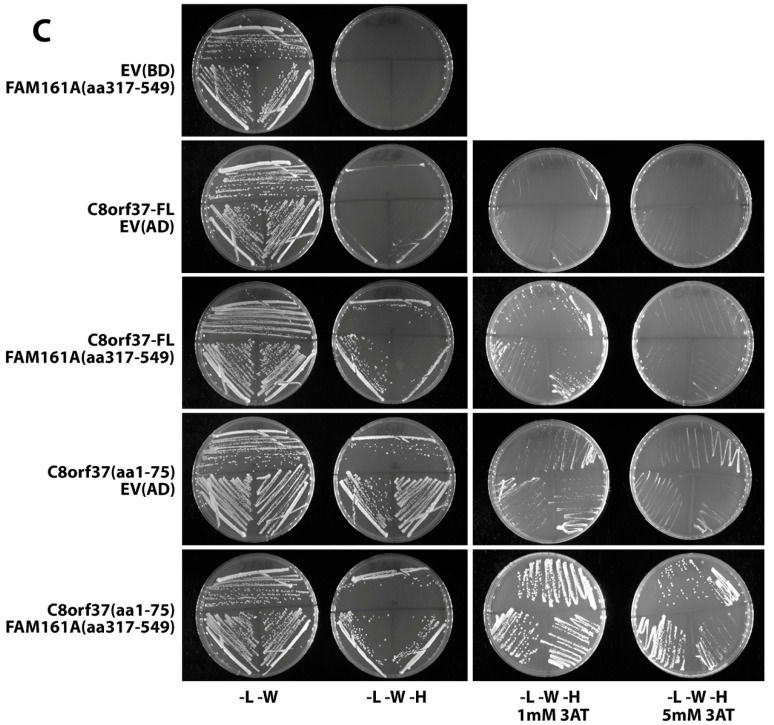
N-terminus of C8orf37 interacts with the UPF0564 domain of FAM161A. Y2H assays were performed to identify the region of interaction on C8orf37 and FAM161A. Transformants were plated at different dilutions on selective medium lacking leucine and tryptophan (-L -W); on medium lacking leucine, tryptophan, and histidine (-L -W -H); on stringent selective medium lacking leucine, tryptophan, and histidine treated with 1mM 3-amino-1,2,4-triazole (-L -W -H +1 mM 3AT); and on medium lacking leucine, tryptophan, and histidine treated with 5mM 3AT (-L -W -H +5 mM 3AT). Schematic (**A**) shows full-length C8orf37, mutant C8orf37, and C8orf37 fragments used in the Y2H experiments. Images in (**B**) show that positive interaction, indicated by colony growth, could be observed between full length C8orf37 (C8orf37–FL) and FAM161A (aa 317–549), C8orf37-R177W and FAM161A (aa 317–549), and C8orf37-Q182R and FAM161A (aa 317–549). No growth was observed between C8orf37 (aa 69–207) and FAM161A. Images in (**C**) show that positive interaction could be observed between the N-terminal fragment of C8orf37 (aa 1–75) and FAM161A (aa 317–549) on -L -W -H plates treated with 1 mM 3AT and 5 mM 3AT, respectively. Abbreviations: EV, empty vector; BD, binding domain; AD, activation domain.

The C-terminal half of C8orf37 is highly conserved across multiple species [6]. Mutations within this region, C8orf37 (R177W) [2,6] and C8orf37 (Q182R) [6], are implicated in Bardet-Biedl syndrome (numbering 21) and retinitis pigmentosa (numbering 64), respectively. Thus, we hypothesized that this region may interact with FAM161A. One-by-one Y2H assays were performed to test this hypothesis. Coding sequences for full-length human C8orf37 (C8orf37-FL), full-length C8orf37 (R177W), and C8orf37 (Q182R), as well as C8orf37 fragment corresponding to residues 69-207 (C8orf37 (aa 69-207)) were used as bait fused to the LexA DNA binding domain and a FAM161A fragment corresponding to aa 317-549 was fused to the GAL4 activation domain. While the yeast transformed with an empty vector and neither C8orf37 nor FAM161A vectors grew in selective growth media, SMAD/SMURF positive control grew in stringent selective growth media lacking leucine, tryptophan, and histidine treated with 1mM 3-amino-1,2,4-triazole (-L -W -H +1mM 3AT) (Figure 5B). As expected, yeast transformed with FAM161A (aa 317–549) and C8orf37-FL grew under stringent selective media. Surprisingly, yeast transformed with FAM161A and either C8orf37 (R177W) or C8orf37 (Q182R) mutants grew under the same conditions as well, indicating that these mutations did not affect interactions between C8orf37 and FAM161A. Further, C8orf37 (aa 69–207)/FAM161A (aa 317–549) did not grow under any stringent conditions. These results indicated that the C-terminal aa 69–207 residues of C8orf37 did not interact with FAM161A. Thus, we tested whether the N-terminal region of C8orf37 (aa 1–75) interacted with FAM161A likewise, with full-length C8orf37 as a positive control. Again, while the yeast transformed with an empty vector and neither full-length C8orf37 nor FAM161A (aa317–549) grew in selective growth media, full-length C8orf37/FAM161A positive control did grow. Yeast transformed with C8orf37 (aa 1–75) and the GAL4 activation domain empty vector grew under selective growth media, suggesting self-activation by aa 1–75 of C8orf37. However, growth of C8orf37 (aa 1–75)/FAM161A (aa 317–549) double-transformed yeast was much more robust in -L-W-H medium treated with both 1 mM 3AT and 5 mM 3AT (Figure 5C). These results indicated that aa 1–75 of C8orf37 can interact with FAM161A.

### 2.4. C8orf37 Interacted with PFAM UPF0564 Domain of FAM161A

**Figure 6:**
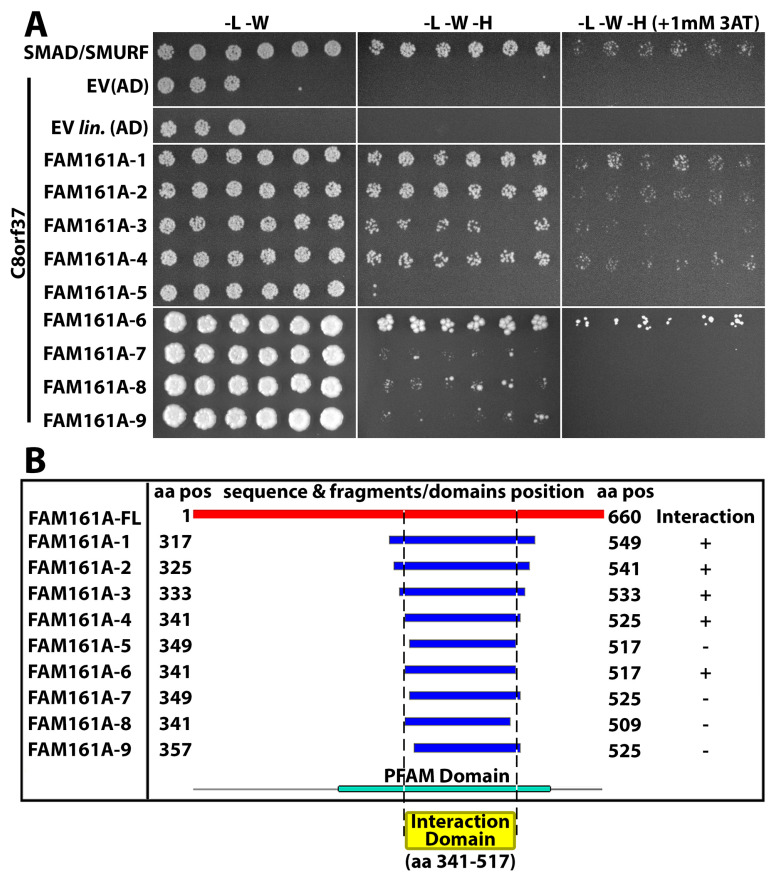
FAM161A amino acid residues 341–517 are necessary for interaction with C8orf37. Interaction domain mapping was performed to identify the minimal region on FAM161A required for C8orf37 interaction. As seen in (**A**), interaction domain mapping was carried out using 9 fragments of FAM161A cloned into the GAL4 activation domain prey vector: aa 317–549 (FAM161A-1), aa 325–541 (FAM161A-2), aa 333–533 (FAM161A-3), aa 341–525 (FAM161A-4), aa 349–517 (FAM161A-5), aa 341–517 (FAM161A-6), aa 349–525 (FAM161A-7), aa 341–509 (FAM161A-8) and aa 357–525 (FAM161A-9). Positive interaction, indicated by colony growth, was observed between FAM161A-1, -2, -3, -4, -6 fragments and C8orf37. No growth was observed in the FAM161A-5, -7, -8, -9/C8orf37 yeast. Chart (**B**) shows a summary of the interaction domain mapping assay. The minimal region of FAM161A necessary for C8orf37 interaction is aa 341-517, which lies within the PFAM UPF0564 domain. Abbreviations: EV, empty vector; EV lin., linearized empty vector; AD, activation domain.

To further define the UPF0564 domain of FAM161A that interacted with C8orf37, coding sequences corresponding to FAM161A fragments aa 317–549 (FAM161A-1), aa 325–541 (FAM161A-2), aa 333–533 (FAM161A-3), aa 341–525 (FAM161A-4), aa 349–517 (FAM161A-5), aa 341–517 (FAM161A-6), aa 349–525 (FAM161A-7), aa 341–509 (FAM161A-8), and aa 357–525 (FAM161A-9) were cloned into the GAL4 activation domain vector and transformed with full-length C8orf37 bait plasmid into yeast. While the yeast transformed with C8orf37 and empty activation domain vectors did not grow in selective growth media, SMAD/SMURF positive control did grow. FAM161A-1, -2, -3, -4, and -6/C8orf37 yeast grew in stringent selective media treated with 1mM 3AT, indicating that aa 341–517 of FAM161A were sufficient for interaction with C8orf37 (Figure 6A). Interestingly, further deleting 8 or 16 residues from the N-terminus (FAM161A-5, -7, and 9) or 8 residues from the C-terminus (FAM161A-8), within aa 341–517, abolished growth (Figure 6A). Figure 6B summarizes the results from this interaction domain mapping experiment. These findings revealed that the minimal domain of FAM161A required for C8orf37 interaction lies between aa 341–517, a region within the UPF0564 domain.

## 3. Discussion

The precise function of C8orf37 in photoreceptor survival is not clear. C8orf37 protein was shown to be present at the base of the primary cilium in hTert-RPE1 cells [6]. In the mouse retina, C8orf37 was also found at the ciliary base and along the ciliary rootlet of photoreceptors [6]. C8orf37 knock-down in zebrafish results in defective formation of the Kupffer’s vesicle, a ciliated developmental organ involved in establishing left–right asymmetry, as well as defects in retrograde melanosome transport [1]. These data suggest that C8orf37 may be involved in the function of the primary cilium. Ciliary expression of C8orf37 was contested in a recent publication [15]. Shariff, et al. [15] propose that C8orf37 is distributed throughout photoreceptor cells excluding the outer segments, as they found that GFP fluorescence in photoreceptors expressing EGFP-C8orf37 fusion protein was widely distributed throughout photoreceptors. Their data showed that overexpressed C8orf37 was localized to photoreceptor cilia in addition to the inner segments and cell bodies. Our results from the marmoset retina showed that C8orf37 was enriched at the ciliary base, present along the axonemes and often co-localized with microtubules in the inner segments of photoreceptors. In addition, C8orf37 was widely expressed throughout the rest of the neural retina, including the photoreceptor cell bodies in the ONL as well as cells in the INL and GCL. The data from Y2H assays, double immunofluorescence staining, and proximity ligation assays strongly supported that C8orf37 interacted with FAM161A. This interaction was not limited to photoreceptor cilia. It was also present in other cell layers of the neural retina. In mouse retina, FAM161A protein is localized at the basal bodies, connecting cilia and inner segments of photoreceptors as well as the OPL, the IPL and the GCL [12,13]. This study showed that, in the marmoset retina, the FAM161A expression pattern was similar to that of the mouse; however, it was also expressed in the ONL and INL. FAM161A associates with microtubules in COS-7, HeLa, hTERT-RPE1, and ARPE-19 cells [12,13]. In the marmoset retina, our data suggested that FAM161A co-localized with microtubules, as well. Thus, C8orf37-FAM161A interaction may play a role in microtubule function. Expression and interaction of C8orf37 and FAM161A in cells other than photoreceptors suggest that they function in other cells, in addition to photoreceptors. Our finding that C8orf37 interacts with FAM161A is consistent with the idea that C8orf37 and FAM161A function, at least in part, as ciliary proteins in photoreceptors.

C8orf37 knockout mice manifest photoreceptor degeneration [15]. The photoreceptors in these mice exhibit defective organization of outer segment discs. Interestingly, outer segment disc defects are often found in rodent models with mutations in outer segment proteins or ciliary proteins, such as peripherin/rds [16,17], GARPs and the beta-subunit of rod cGMP-gated channels [18], protocadherin 21 [19], RP1 [20], RPGR-interacting protein [21], and prominin 1 [22]. Photoreceptors in FAM161A mutant mice exhibit shortened cilia with malformed discs in the outer segment [23]. Disorganization of the outer segment discs in C8orf37 knockout mice is consistent with the hypothesis that C8orf37 plays a role in photoreceptor cilia or outer segments.

**Figure 7:**
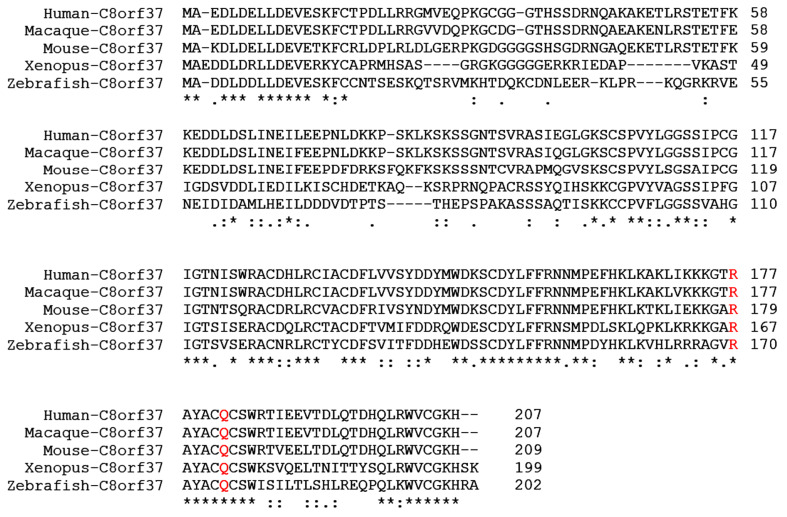
Alignment of C8orf37 homologs. Alignment of human, macaque, mouse, *Xenopus laevis*, and zebrafish homologs was performed via CLUSTAL O (1.2.4). Amino acid residues 1–17 and 100–207 of C8orf37 are highly conserved between human, macaque, mouse, Xenopus and zebrafish. The C8orf37 (R177W) and C8orf37 (Q182R) mutations are indicated in red. Fully conserved residues are denoted by an asterisk (*).

Alignment of C8orf37 proteins from several species indicates that ~20 residues from the N-terminus and aa 100–207 of the C-terminal half are highly conserved (Figure 7). In addition to splicing and nonsense mutations that may abolish the function of C8orf37, the Q182R mutation is found in RP64 [6] while the R177W mutation is found in CORD16 [6] and BBS21 [2]. These residues are conserved in human, macaque, mouse, and Xenopus, as well as zebrafish C8orf37 orthologues (Figure 7), suggestive of their importance in C8orf37 protein function. Our Y2H assay results indicated that these mutations did not disrupt C8orf37-FAM161A interactions. In fact, our results indicated that the C-terminal aa 69–207 region of C8orf37 was not able to interact with FAM161A. Therefore, the pathogenicity of these two mutations is likely not dependent upon C8orf37-FAM161A interaction. Although no pathogenic mutations have been identified affecting the first 75 amino acid residues at the N-terminal end of C8orf37, our direct 1-by-1 Y2H assays revealed that the N-terminal aa 1–75 region on C8orf37 was sufficient and required for its interaction with FAM161A. Characterizing the function of these highly conserved N-terminal and C-terminal regions on C8orf37 will necessitate further investigation.

Human FAM161A encodes a protein comprising 660 residues (isoform 1). Our interaction domain mapping analyses have limited the minimal interaction region to within aa 341–517. Several pathogenic mutations, p.Arg437* [10,24,25,26,27,28], p.Thr452Serfs*3 [11,29,30], p.Trp488* [26], and p.Lys501Valfs*4 [24,27,28,31], have been identified within this region. Additionally, nonsense mutations occurring in residues N-terminal to aa 341–517, p.Lys227Asnfs*4 [32], p.Arg229* [10], p.Asp261Valfs*39 [32], p.Lys315* [33] and p.Arg335* [34,35], have been reported. These mutations all result in disruption of this minimal interaction domain on FAM161A. The aa 341–517 region of FAM161A lies within the PFAM UPF0564 domain which spans aa 234–592. This highly conserved domain is predicted to bind to microtubules [13] as well as proteins linked to retinal degeneration and ciliary trafficking, such as CEP290, OFD1, and LCA5 [12,36,37,38,39,40]. Thus, C8orf37-FAM161A interactions may play a crucial role in protein sorting or trafficking through cilia. Future studies will further define the molecular interactions between C8orf37 and FAM161A and determine how this interaction may contribute to photoreceptor survival.

## 4. Materials and Methods

### 4.1. Yeast Two-Hybrid

The Y2H assays were performed at Hybrigenics Services SAS (Evry, France) on a fee-for-service basis. Coding sequence of full-length human C8orf37 (NM_177965.3) was cloned into pB27 derived from the pBTM116 vector [41] with the LexA DNA binding domain (DBD) as a LexA–bait fusion. The bait library was constructed from a human retina cDNA library cloned into the pP6 vector. In total, 50.5 million clones were screened.

The direct 1 by 1 interaction assays were performed using coding sequences of full- length human C8orf37, C8orf37 (R177W), C8orf37 (Q182R), C8orf37 (aa 1–75), and C8orf37 (aa 69–207) cloned into pB27 derived from the pBTM116 vector [41] with the LexA DNA binding domain (DBD) as a LexA–bait fusion. A FAM161A (NM_032180.3) coding fragment corresponding to amino acids 317-549 was extracted from the ULTImate Y2H^TM^ screening of full-length C8orf37 with a human retina cDNA library and cloned into the pP6 vector derived from pGADGH [42] with the GAL4 activation domain (AD). Next, these constructs were transformed in L40deltaGAL4 (mata) and YHGX13 (Y187 ade2-101::loxP-kanMX-loxP, matα) yeast haploid cells, respectively. Diploid cells were obtained by mating both yeast strains as described in [43]. Empty vectors (EV) pP7 and pB27 were used as bait and prey negative controls, respectively; SMAD and SMURF interaction was used as a positive control [44]. Several dilutions (undiluted 5 × 10^7^ cells, 10^−1^, 10^−^^2^, 10^−3^) of the diploid yeast cells expressing bait and prey constructs were grown on selective media lacking tryptophan and leucine (DO-2, -L -W) and selective media lacking tryptophan, leucine, and histidine (DO-3, -L -W -H). Varying concentrations of 3-AT were added to the DO-3 plates to increase stringency. 

For the interaction domain mapping assay, coding sequences corresponding to fragments of FAM161A (aa 317–549, aa 325–541, aa 333–533, aa 341–525, aa 349–517, aa 341–517, aa 349–525, aa 341–509, aa 357–525) were amplified by PCR and transformed in yeast with the C8orf37 bait plasmid and linearized pP7 prey plasmid. Recombinant clones were grown and selected on media lacking tryptophan and leucine (DO-2). Six diploids for each fragment were selected and tested for interaction with C8orf37 by growth assay on medium lacking tryptophan, leucine, and histidine (DO-3).

### 4.2. Transfection of HEK293 Cells

HEK293 cells were maintained in Dulbecco’s Modified Eagle Media (ThermoFisher Scientific, Grand Island, NY, USA) supplemented with 10% fetal bovine serum. Transient transfection of pCMV-C8orf37-turboGFP (C8orf37-tGFP, OriGene Technologies, Inc., Rockville, MD, USA 20850, cat#RG222816) and pCMV-FAM161A-c-Myc (Origene Technologies, cat#RC229326) expression plasmids was performed using the calcium phosphate method (Rodriguez and Flemington, 1999). The day before transfection, 80% confluent cells were split 1:10 and plated onto 8-well tissue culture chamber slides (Ibidi USA, Inc., Fitchburg, WI, USA, cat#80841). The following day, 0.5 mL 1 × HBS (0.5% HEPES, 0.8% NaCl, 0.1% dextrose, 0.37% KCl, 0.01% Na_2_HPO_4_(7H_2_O); pH = 7.05), 30 µL 2.5 M CaCl_2_ and 30 µg DNA were mixed at room temperature. A volume of 8 μL of the transfection mix was then added to the cells in a dropwise fashion to each well. The cells were analyzed 48 h post-transfection.

### 4.3. Immunofluorescence Staining

Common marmoset eye samples were obtained at the Central Institute for Experimental Animals (CIEA), Kawasaki, Kanagawa, Japan. All handling of animals was in accordance to the guidelines published by the Institute for Laboratory Animal Research (Guide for the Care and Use of Laboratory Animals) and the US Public Health Service (Public Health Service Policy on Humane Care and Use of Laboratory Animals). All animal experiments at CIEA were approved by the Institutional Animal Care and Use Committee of the Central Institute for Experimental Animals (CIEA approval number: 18031A, 19033A).

Marmoset retina were fixed with 4% paraformaldehyde, embedded in OCT media, cryo-sectioned, and mounted onto Fisherbrand Superfrost Plus slides (ThermoFisher Scientific, Grand Island, NY, USA, cat#12-550-15). Cells cultured on chamber slides were fixed in 4% paraformaldehyde for 10 min and permeabilized with 0.1% Triton X-100 in 0.1 M phosphate buffer (PB). For immunofluorescence staining, the samples were blocked with 3% bovine serum albumin (BSA) in 0.1M PB for one hour at room temperature. Primary antibodies rabbit anti-C8orf37 (Novus, cat#NBP1-93892, 1:300), rabbit anti-FAM161A (Biorbyt LLC, St. Louis, MO, USA, cat#orb2214, 1:300), mouse anti-acetylated α-tubulin (Sigma-Aldrich, St. Louis, MO, USA, cat#T6793, 1:100), and rabbit anti-c-Myc (Sigma-Aldrich, cat#3956, 1:300) were applied overnight at 4 °C in a humidified chamber. The samples were then washed with 0.1M PB. The appropriate fluorophore, either FITC- or RITC-conjugated goat anti-rabbit IgG (Jackson ImmunoResearch Laboratories, Inc., West Grove, PA, USA, 1:300) or goat anti-mouse IgG (Jackson ImmunoResearch Laboratories Inc., 1:300) was applied for 2 h at room temperature in a humidified chamber. After washing, the samples were counterstained with 4′,6-diamidino-2-phenylindole (DAPI) to visualize nuclei. Coverslips were mounted using VECTASHIELD^®^ mounting medium (Vector Laboratories, Burlingame, CA, USA, Cat# H-1000). A Zeiss confocal microscope system (Zeiss LSM 780) was used to obtain confocal images.

### 4.4. Proximity Ligation Assay 

For proximity ligation assay on marmoset retinal sections, rabbit antibodies against C8orf37 and FAM161A were conjugated with the Duolink In Situ Probemaker Plus (Sigma-Aldrich; cat#DUO92009-1KT) and the Duolink In Situ Probemaker Minus (Sigma-Aldrich; cat#DUO92010-1KT) kits, respectively. Proximity ligation assay was performed using the Duolink In Situ Detection Reagents Red kit (Sigma-Aldrich, cat# DUO92008) according to the manufacturer’s recommendation. A Zeiss confocal microscope system (Zeiss LSM 780) was used to obtain images. To quantify PLA signals, images were exported to ImageJ “https://imagej.nih.gov/ij/ (accessed on 1 September 2015)”. Each image was divided into multiple regions corresponding to the photoreceptor outer segments (OS), photoreceptor inner segments (IS), outer nuclear layer (ONL), outer plexiform layer (OPL), inner nuclear layer (INL), inner plexiform layer (IPL), and ganglion cell layer (GCL). Within each layer, the total number of PLA puncta was manually counted in ImageJ and normalized to a 100 µm retina.

For proximity ligation assay of epitope-tagged human C8orf37 and human FAM161A, transfected cells were fixed with 4% paraformaldehyde 48 h post-transfection. Rabbit anti-c-Myc and mouse anti-turboGFP (tGFP) (Origene Technologies, cat#TA150041, 1:500) primary antibodies were incubated with the slides in a procedure identical to immunofluorescence staining. After washing, proximity ligation assays (PLA) were performed using the Duolink In Situ Red Starter Kit Mouse/Rabbit (Sigma-Aldrich; Cat#DUO92101) according to the manufacturer’s recommendations. Confocal images were obtained, as above. To quantify PLA puncta all images were exported to ImageJ for processing. The ImageJ manual thresholding function was used to adjust each image to eliminate background fluorescence in the PLA color channel. Following thresholding, PLA puncta were automatically counted using the “Analyze Particles” function in ImageJ with the minimum particle size set at 0.1 µm^2^. For each image, the number of PLA puncta was normalized to the number of nuclei visible. The experiments were performed in triplicate with the average number of PLA puncta per cell within each replicate consisting of results from five images.

## Figures and Tables

**Table 1:** Prey proteins identified in Y2H screen.

Protein Name (Gene Name)	Confidence of Interaction *
C22orf19 (THOC5)	Moderate
CASP8-associated protein 2 (CASP8AP2)	Moderate
Chromodomain helicase DNA-binding protein 3 (CHD3)	Good
Cone–rod homeobox (CRX)	Moderate
Death domain-associated protein (DAXX)	Moderate
Family with sequence similarity 161, member A (FAM161A)	Moderate
Homeodomain-interacting protein kinase 2, variant 2 (HIPK2)	Very High
Heat shock transcription factor 2 (HSF2)	Moderate
Mitochondrial ribosomal protein S6 (MRPS6)	Moderate
Plectin (PLEC)	Moderate
Protein tyrosine kinase 2 Beta (PTK2B)	Good
RING finger protein 111 (RNF111)	Moderate
Synaptotagmin-binding cytoplasmic RNA- interacting protein (SYNCRIP)	Moderate
Zinc finger protein 106 (ZNF106)	Moderate
Non-receptor tyrosine kinase, Src (nRTK)	Moderate
Ankyrin repeat domain 11 (ANKRD11)	Moderate
RNA-binding fox-1 homolog 1 (RBFOX1)	Moderate
N-acetylneuraminate pyruvate lyase (NPL)	Moderate
Thioredoxin reductase 2 (TXNRD2)	Moderate
DEAD box helicase 17 (DDX17)	Moderate
SWI/SNF-related matrix-associated actin-dependent regulator of chromatin subfamily A containing DEAD/H box 1 (SMARCAD1)	Moderate
Metastasis-associated lung adenocarcinoma transcript 1 (MALAT1/NEAT2)	Moderate
Calcium/calmodulin-dependent protein kinase type 1D (CAMK1D)	Moderate
Phosphodiesterase 4D (PDE4D)	Moderate
Tripartite motif-containing 37 (TRIM37)	Moderate
FSHD region gene 1 (FRG1)	Moderate
Synapsin III (SYN3)	Moderate

* Confidence of interaction is the predicted biological score (PBS) calculated as described in Formstecher et al., 2005 [14].

## Supplementary Materials

The following supporting information can be downloaded at: https://www.mdpi.com/article/10.3390/ijms231912033/s1.
