# Supplementary material for: Interactions between C8orf37 and FAM161A, Two Ciliary Proteins Essential for Photoreceptor Survival
Source: Int J Mol Sci. 2022 Oct 10;23(19):12033. doi: 10.3390/ijms231912033 (PMC9570145; doi:10.3390/ijms231912033)

Supplementary Figure S1. Anti-C8orf37 specifically labels C8orf37.

HEK293 cells were transfected with pCMV-C8orf37-tGFP expression construct. The transfected cells were double-stained with tGFP (green) and C8orf37 (red) antibodies (A-C). Nuclei were counterstained with DAPI (blue). Western blot was performed using lysates from pCMV-C8orf37-tGFP transfected HEK293 cells (D).

(A-C) Anti-C8orf37 immunoreactivity (red) overlapped with anti-tGFP immunoreactivity (green) in transfected HEK293 cells. Scale bar in C: 7μm for A-C.

(D) Both anti-tGFP and anti-C8orf37 labeled the expected 50 kDa C8orf37-tGFP band on Western blot.

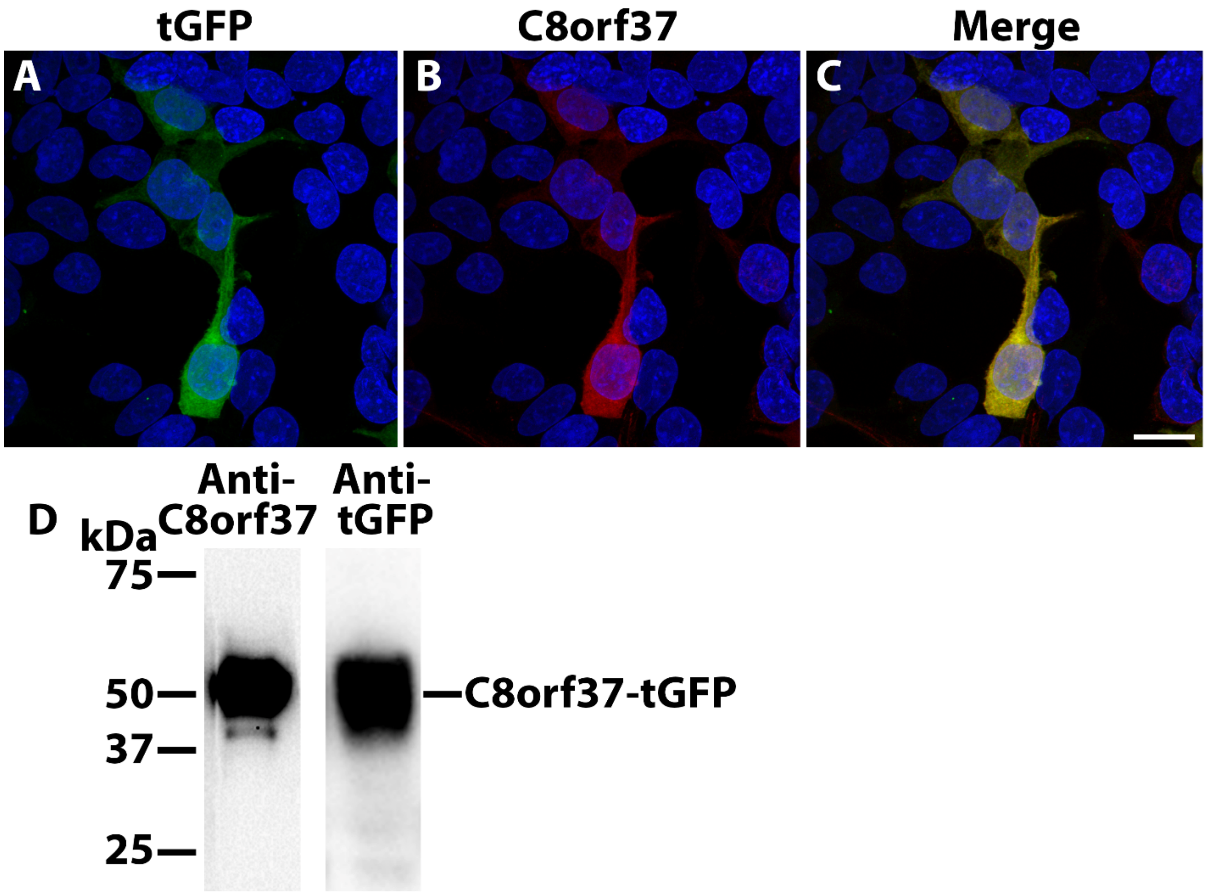

Supplement: Supplementary file 1 [file ijms-23-12033-s001.zip › ijms-1945619-supplementary.pdf]
